# Polyploidy and mTOR signaling: a possible molecular link

**DOI:** 10.1186/s12964-024-01526-9

**Published:** 2024-03-27

**Authors:** Debopriya Choudhury, Dhruba Ghosh, Meghna Mondal, Didhiti Singha, Ramesh Pothuraju, Pushkar Malakar

**Affiliations:** 1 Department of Biomedical Science and Technology, School of Biological Sciences, Ramakrishna Mission Vivekananda Educational Research Institute (RKMVERI), Kolkata, India; 2 Cancer Research Program, Rajiv Gandhi Center for Biotechnology (RGCB), Thiruvananthapuram, 695014 Kerala India

**Keywords:** Polyploidy, mTOR signaling, Cancer, Diabetes, Aging, mTORC1, mTORC2

## Abstract

Polyploidy is typically described as the condition wherein a cell or organism has more than two complete sets of chromosomes. Occurrence of polyploidy is a naturally occurring phenomenon in the body’s development and differentiation processes under normal physiological conditions. However, in pathological conditions, the occurrence of polyploidy is documented in numerous disorders, including cancer, aging and diabetes. Due to the frequent association that the polyploidy has with these pathologies and physiological process, understanding the cause and consequences of polyploidy would be beneficial to develop potential therapeutic applications. Many of the genetic and epigenetic alterations leading to cancer, diabetes and aging are linked to signaling pathways. Nonetheless, the specific signaling pathway associated with the cause and consequences of polyploidy still remains largely unknown. Mammalian/mechanistic target of rapamycin (mTOR) plays a key role in the coordination between eukaryotic cell growth and metabolism, thereby simultaneously respond to various environmental inputs including nutrients and growth factors. Extensive research over the past two decades has established a central role for mTOR in the regulation of many fundamental cellular processes that range from protein synthesis to autophagy. Dysregulated mTOR signaling has been found to be implicated in various disease progressions. Importantly, there is a strong correlation between the hallmarks of polyploidy and dysregulated mTOR signaling. In this review, we explore and discuss the molecular connection between mTOR signaling and polyploidy along with its association with cancer, diabetes and aging. Additionally, we address some unanswered questions and provide recommendations to further advance our understanding of the intricate relationship between mTOR signaling and polyploidy.

## Introduction

**Fig. 1:**
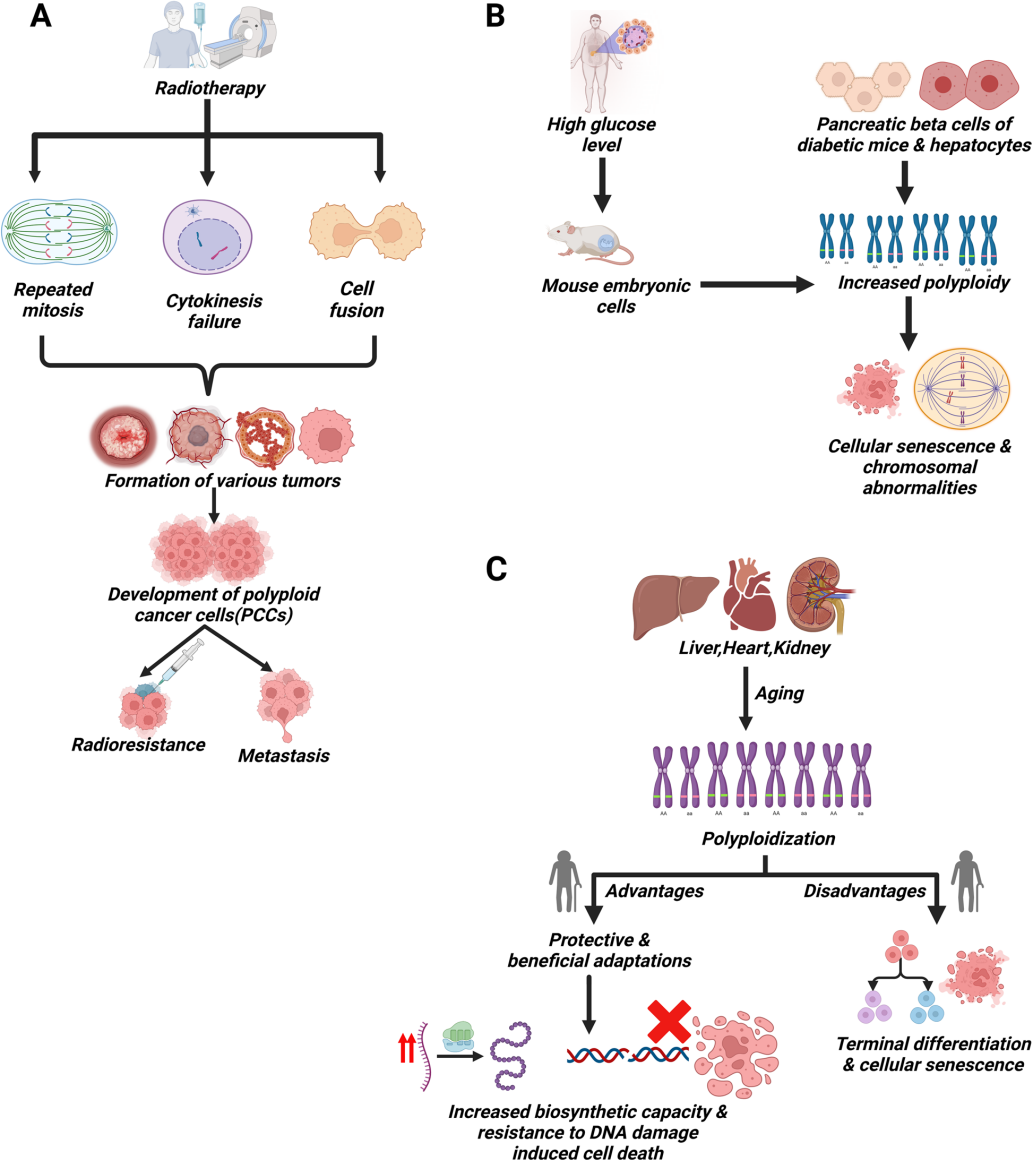
Association of polyploidy with cancer, diabetes and aging: (**A**) Illustration depicting the genesis and function of polyploid giant cancer cells (PGCC) in cancer. **B** The link between polyploidy and diabetes. **C** Overview of the connection and significance of polyploidy in the aging process

Polyploidy is a condition in which a cell or organism has more than two complete sets of chromosomes [1]. For instance, humans can exhibit triploidy with 69 chromosomes or tetraploidy with 92 chromosomes [2]. The formation of polyploid cells in a diploid organism can occur via three primary mechanisms: cell fusion, endoreplication, and other abnormalities that lead to an unsuccessful cell cycle [3–5]. Moreover, polyploidy can emerge from multinucleate conditions due to unsuccessful cytokinesis or cell fusion [6]. Polyploid giant cells, frequently characterized by the presence of many nuclei, originate from malignant cell lines and malignancies [6, 7]. In addition to cancer, polyploidy has also been associated with aging and diabetes (Fig. 1). Studies have demonstrated that polyploidization rises with age in certain mammalian organs, such as liver, brain, heart and eye [8–10]. Furthermore, diabetic mice exhibit an elevated occurrence of polyploidization in pancreatic β-cells [11]. The transition from diploidy to polyploidy is a natural process that occurs during the development and differentiation processes in an organism under normal physiological conditions [10]. Polyploidization has further been regarded as a frequent occurrence in evolution [12]. mTOR signaling plays a very important role in generating characteristic features of polyploid cells.

**Fig. 2:**
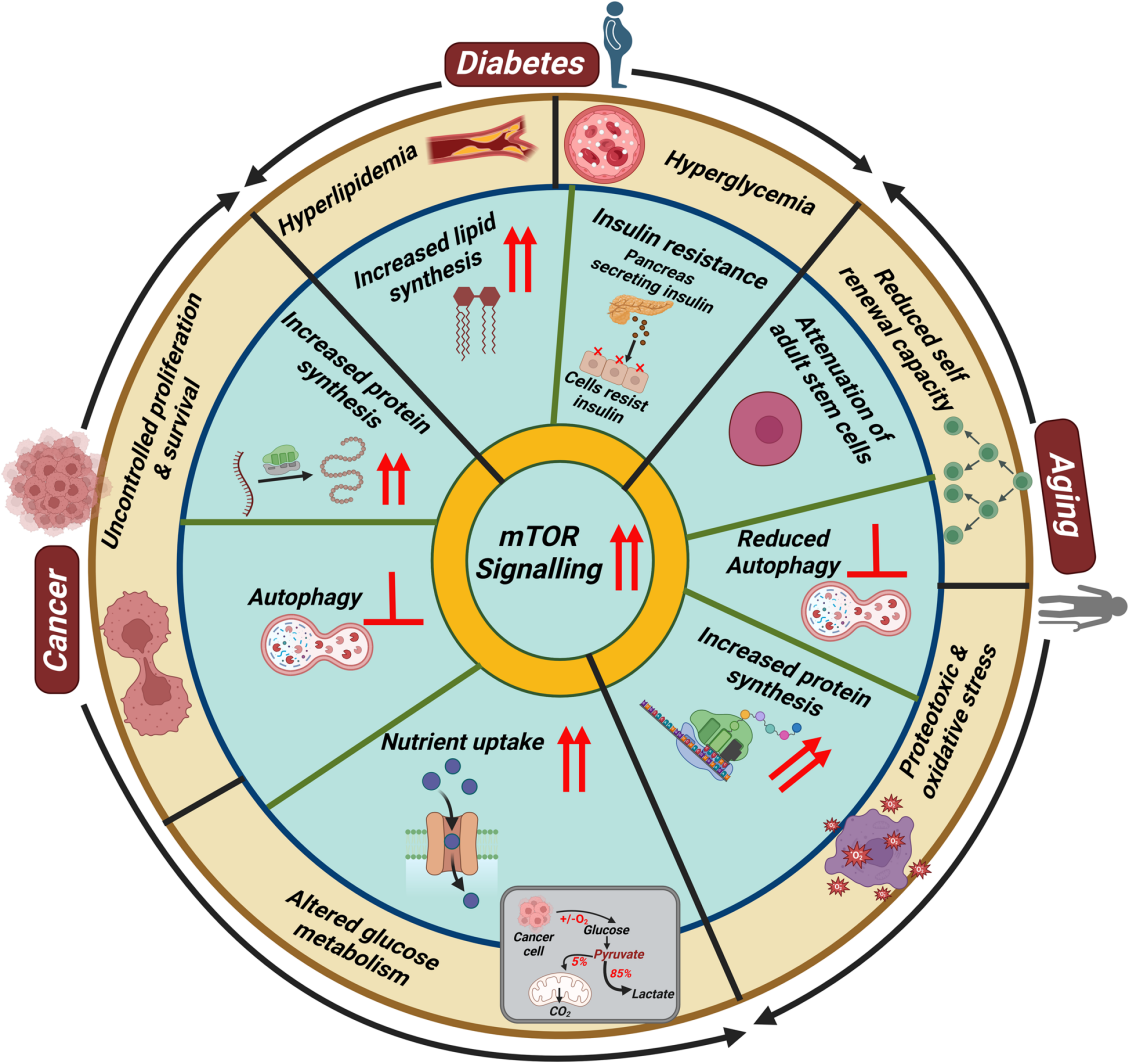
Association of the mTOR signaling pathway with cancer, diabetes and aging: Illustrative depiction of the interplay and functional relevance of mTOR signalling in cancer, diabetes and aging

mTORpathway plays a central role in the regulation of multiple fundamental cellular processes, that range from protein synthesis to autophagy [13]. Dysregulated mTOR signaling has been implicated in the progression of cancer, diabetes and the aging process (Fig. 2) [13]. mTOR signaling has frequently been affected in human cancers. Due to the involvement of the mTOR pathway in the regulation of essential processes such as cell cycle, proliferation, growth, survival, protein synthesis and glucose metabolism, mTOR is closely linked to cancer [14]. The mTOR signaling pathway has frequently been found to be dysregulated in individuals with diabetes as well. This dysregulation has been triggered by multiple occurrences such as insulin resistance, elevated triglyceride levels, lipid metabolism, ketone production, glucose metabolism and cholesterol metabolism [13]. mTOR generates two distinct multiprotein complexes, namely mTORC1 and mTORC2, which serve diverse functions [13]. mTOR signaling pathway comprises of several protein partners that regulate multiple cellular processes such as metabolism, protein synthesis and cell development [13]. Both Type 1 and Type 2 diabetes (T2D) have been associated with mTORC1, suggesting that it plays a role in the development of these diseases [13, 14]. The most popular treatment for T2D, which is metformin, controls mTORC1 activity [15]. Furthermore, genetic evidence has established the association between mTOR signaling and aging process as well [14]. According to genetic studies, reduced mTOR signaling, increases cellular longevity [14]. It has widely been hypothesized that the positive benefits of calorie restriction on life span have been imposed by lowered mTORC1 signaling due to the crucial role that mTORC1 plays in the integration of nutrition with insulin signaling [16]. There is a strong correlation between the hallmarks of polyploidy and mTOR signaling. This review focuses on the molecular connection between polyploidy and mTOR signaling.

### Polyploidy in cancer

Human malignancies have been found to exhibit unscheduled tetraploidy [17, 18]. Tetraploidy is a form of polyploidy characterized by the presence of four sets of chromosomes within a single cell [18]. Tetraploidy has been observed in the initial phases of colon cancer, breast cancer, Barrett’s oesophagus and cervical cancer [18]. An elegant study has demonstrated that tetraploid cells which result from the failure of cytokinesis have the ability to induce tumor formation in mice lacking p53 gene [19]. Tetraploid cells have been found to develop into aneuploid cells when cultured in a laboratory setting [18]. Aneuploidy, a significant characteristic of cancer, denotes the existence of an atypical quantity of chromosomes or segments of chromosomes [18]. Tetraploidy is the metastable intermediary state that exists between normal diploidy and malignant aneuploidy [18]. Polyploidy can lead to alterations in genetic material that enhance the likelihood of producing advantageous mutations [18]. Although polyploid cells in a diploid organism can provide immediate benefits, polyploidy may also entail long-term drawbacks or liabilities [20–22]. During division, polyploid cells may allow for maximum metabolic efficiency due to the presence a reduced number of stable genomes [20, 23]. The possible expense of producing polyploid cells is corroborated by the recent identification of a tetraploidy checkpoint that kills tetraploid cells by apoptosis [24, 25]. It is fascinating that the tetraploidy checkpoint relies on the primary pathways of p53 and Rb that are implicated in human cancer [25]. These findings further substantiate the need to reassess the long-standing idea that a tetraploid intermediate likely plays a significant role in the development of genomic instability in certain cancers.

Polyploidy in cancer is not limited to tetraploid cells but also extends to polyploid giant cancer cells (PGCCs) [7]. Influences such as hypoxia, chemotherapeutic medicines and radiation, can induce the formation of PGCCs by altering the cell cycle and modulating the expression of fusion associated proteins (Fig. 1A) [26–28]. PGCCs are present in a range of solid malignant tumors, including melanoma, urothelial cancer, kidney cancer, breast cancer, ovarian cancer, pancreatic cancer and prostate cancer [7]. A high number of poorly differentiated tumors reportedly have a significant presence of PGCCs, exceeding the quantities found in well-differentiated and less aggressive tumors [29]. Higher abundance of PGCCs is observed in lymph node metastatic foci than primary tumor tissues [29]. Elevation of PGCCs has been observed in the tumor tissue after treatment such as radiotherapy and/or chemotherapy as compared to before treatment tumor tissue [29]. Recent evidence has demonstrated that PGCCs exhibit characteristics of cancer stem cells (CSCs) and undergo asymmetric division to generate new cells [30, 31]. These newly formed cells exhibit markers that are associated with the process of epithelial-mesenchymal transition, which in turn aids in their ability to metastasize [28, 29]. The CSC-like properties that are found in PGCCs aid in the differentiation of these cells into non-cancerous cells. For example, PGCCs can be induced to undergo differentiation and thereby produce erythrocytes that show high affinity for oxygen. This adaptation in turn allows the PGCCs to survive extreme low oxygen condition [32, 33]. Polyploidy has been associated with multiple aspects of cancer development and maintenance, including the ability to resist drugs [7].

### Polyploidy in diabetes

Increased polyploidization has been observed in the pancreatic β-cells of diabetic mice [11, 34, 35]. People with insulin-independent diabetes has a notably elevated proportion of polyploid nuclei in their islets [36]. Individuals with insulin-dependent diabetes also have a much higher proportion of polyploid nuclei in their islets than the control subjects [36]. Hepatocytes from adult, nonobese diabetic mice show changes in polyploidization when compared to non-diabetic control mice of the same age [37, 38]. Cellular senescence has been reported in diabetic islet cells as well as in PGCCs [39]. This implicates possible roles of polyploidy in inducing senescence which is observed in diabetic islets. In addition, polyploid cells have also been observed in mouse embryonic cells that are cultured under diabetic conditions (Fig. 1B) [40]. In diabetic condition, glucose and ketone bodies, which are known to have physiological significance, reportedly induce polyploidy in cultured mouse embryonic cells [40]. A study involving mouse model has demonstrated that excess glucose intake results in an accelerated β-cell polyploidization [11]. Polyploidy is known to be the precursor of chromosomal anomalies that have been observed in the embryos of diabetic mice (Fig. 1B) [40]. The molecular connection between polyploidy and chromosomal instability has not yet been established in the context of diabetes. It has been observed that liver plays a pivotal role in diabetes [41]. It has also been determined that hepatic cells are known to undergo polyploidy [3, 10]. However, the molecular connection between hepatic polyploidy and diabetes is yet to be established. Detailed investigation is required to unravel this yet uncovered connection which in turn would answer the key questions in relation to polyploidy and diabetes.

### Polyploidy in aging

Polyploidization in mammalian organs has been shown to increase with age, specifically in the organs of liver, brain, heart and eyes (Fig. 1C). In the mammalian liver tissue, a substantial population of hepatocytes exists as polyploid cells, and the process of polyploidization is notably amplified during the aging process [10]. The proportion of polyploidy also increases with the aging process involving as much as 90% of the hepatocytes in mice and 40% in humans [10]. Polyploidization in hepatocytes has been shown to be regulated by centrosomes and antiapoptotic protein Mcl-1 [42–44]. It has been observed that centriole signaling through PIDDosome–p53 axis is required to restrict hepatocyte polyploidy and maintain liver integrity [42]. In addition, it has further been shown in vivo that Mcl-1 deficiency leads to polyploidy generation in hepatocytes [43].

Polyploid hepatocytes are an important source of liver regeneration under stressful conditions [45]. Polyploid hepatocytes repeatedly divide to maintain normal turnover of hepatocytes during aging [46]. Gene set enrichment analysis revealed that genes related to immune responses are commonly downregulated in polyploid cells, whereas genes associated with mitochondrial functions such as lipid and fatty acid metabolism are commonly upregulated in polyploid cells compared to diploid cells, suggesting the regulation of functional properties of the liver by polyploidy [46]. Hepatocyte polyploidization, which is generally considered as an indicator of terminal differentiation and cellular senescence, has been found to be related to the dysfunction of insulin and p53/p21 signaling pathways (Fig. 1C) [47].

In regard to function of polyploidy in aging, one school of thought is that the polyploid cells that build up with age, are a form of damage and dysfunction linked to cellular senescence and could play a part in the deterioration and disease that come along with old age [48–50]. Another school of thought is that polyploidy might be a protective, beneficial adaptation that emerges in the damaged environment of aged tissues (Fig. 1C) [51]. It has also been speculated that elevated levels of polyploidy with age might aid in the increase of cellular biosynthetic capacity, which in turn protect the cells from the deleterious effects of DNA damage that is lethal to the diploid cells (Fig. 1C) [52]. An Increased incidence of polyploidization with aging has been observed in the brain cells of *Drosophila* [53]. In a recent study performed on *Drosophila*, it has been speculated that increased polyploidy occurs in response to tissue damage to maintain organ size [53]. This mechanism is likely a physiological strategy that deals with the damaged portion in an aged brain where very limited cell division occurs [53]. In *Drosophila* model, it has been shown that polyploid cells arise from cell fusion, and contribute in the decline of the biomechanical fitness of the organism with the increase of age [53].

Polyploidy contributes to aneuploidy [20]. Aneuploidy typically has negative effects on cellular fitness, often resulting in apoptosis or cellular senescence [54]. Senescent cells increase in number with age, and this might contribute to tissue disorders associated with aging by compromising functionality and reducing the regenerative potential **(**Fig. 1C**)** [55]. The secretion of various pro‐inflammatory proteins from senescent cells which is referred to as senescence‐associated secretory phenotype (SASP), promotes both tissue disorders and aging phenotypes [56]. In a phosphovimentin deficient mouse model, an unforeseen connection between tetraploidy and aging has been observed [57]. It has been found that cytokinetic failure results in the generation of tetraploid cells, which later develop into aneuploid cells, ultimately promoting premature aging [57]. One idea is that as people get older, more senescent cells build up because they are produced in higher abundance and are not getting rid of themselves as quickly as they should [55]. Accumulation of senescent cells could lead to tissue disorders by rendering tissues less functional and less capable of self-healing [9, 58]. Increased tetraploidy of cardiomyocytes and the resultant decrease in their regeneration is a phenotype related to cellular senescence [9, 58]. The association between tetraploidy and aging, as well as the connection between aneuploidy and aging, have recently been emerging which calls for further investigation. With regard to regulation of tetraploids in aging, it has been shown that BAX/BAK, pro-apoptotic proteins of Bcl-2 family members, prevents the induction of a tetraploidization-associated senescence program [59]. To gain deeper insights into these relationships, it is essential to conduct a comprehensive characterization of tetraploidy during physiological aging, and uncover the underlying causative factors. Simultaneously, investigations utilizing animal models will aid in unravelling the relationship between tetraploidy and aging.

### Mechanisms for generation of polyploidy in cancer cell

#### Mitotic cycle to endocycle

Polyploid cells can arise from different processes which are generally divided into two categories: cell fusion (cell–cell fusion) and endocycling [8]. Endocycle is a process in cell cycle wherein DNA replication is repeated without mitosis or cytokinesis [8]. It has been documented that the mechanism of endocycle leads to polyploidy [4]. Endoreplication, which is a type of endocycling, refers to the replication of DNA during the S phase of the cell cycle that takes place without subsequent completion of mitosis and/or cytokinesis [60].

#### Cell–cell fusion

Cell–cell fusion is a process where two or more cells join together by fusing their membranes [61]. It is crucial in multiple processes such as fertilization, organ development, immune response and regeneration [62]. Cell fusion is further implicated in the generation of polyploidy cells at the time of aberrant cell fusion [63].

#### Multinucleation

The occurrence of polyploidy can result from multinucleate conditions following unsuccessful cytokinesis or cell fusion [6]. Polyploidization observed in cardiomyocytes is often associated with multinucleation [58]. PGCCsare often found to possess giant multinucleated cells [7]. Multinucleation is associated with the generation of polyploid giant cells from nasal mucosal neoplasm when infected with Epstein-Barr virus [64]. Continuous expression of latency-associated nuclear antigen in different cell lines leads to multinucleated phenotypes that are linked to the formation of PGCCs [65]. Tax is a pleiotropic oncoprotein that is needed for viral replication to function. It often causes cells to become multinucleated and polyploid [64]. The formation of solid tumors by a single multinucleated cancer cell has already been established [66]. Multiple nuclei and failed cell division are linked to human papilloma virus infection, which is known to cause the formation of PGCCs [67]. Endomitosis is an incomplete form of mitosis that does not culminate in cell division but gives rise to multinucleated giant cells [68]. Abortive cell division reportedly often results in a multinucleated cell [67].

#### Abortive cell cycle

Interrupting the control of the cell cycle is one way that polyploidy might happen. When cells keep making DNA without stopping by for cytokinesis, polyploid cells are developed [69]. Endoreplication, cytokinesis failure and mitotic slippage are three examples of failed cell cycles that result in polyploidy [10]. A cell cycle wherein mitosis has started and progresses normally through the end of anaphase but is not completed results in a cell with increased ploidy [70]. Melanomas and their precursors, specifically melanocytes, are exposed to UV light because of their anatomic location in the dermis. This in turn results in DNA damage and stress due to ROS production, which further leads to the formation of polyploid cells, especially when mitosis or cell division fails [71].

### Hallmarks of polyploid cells

**Fig. 3:**
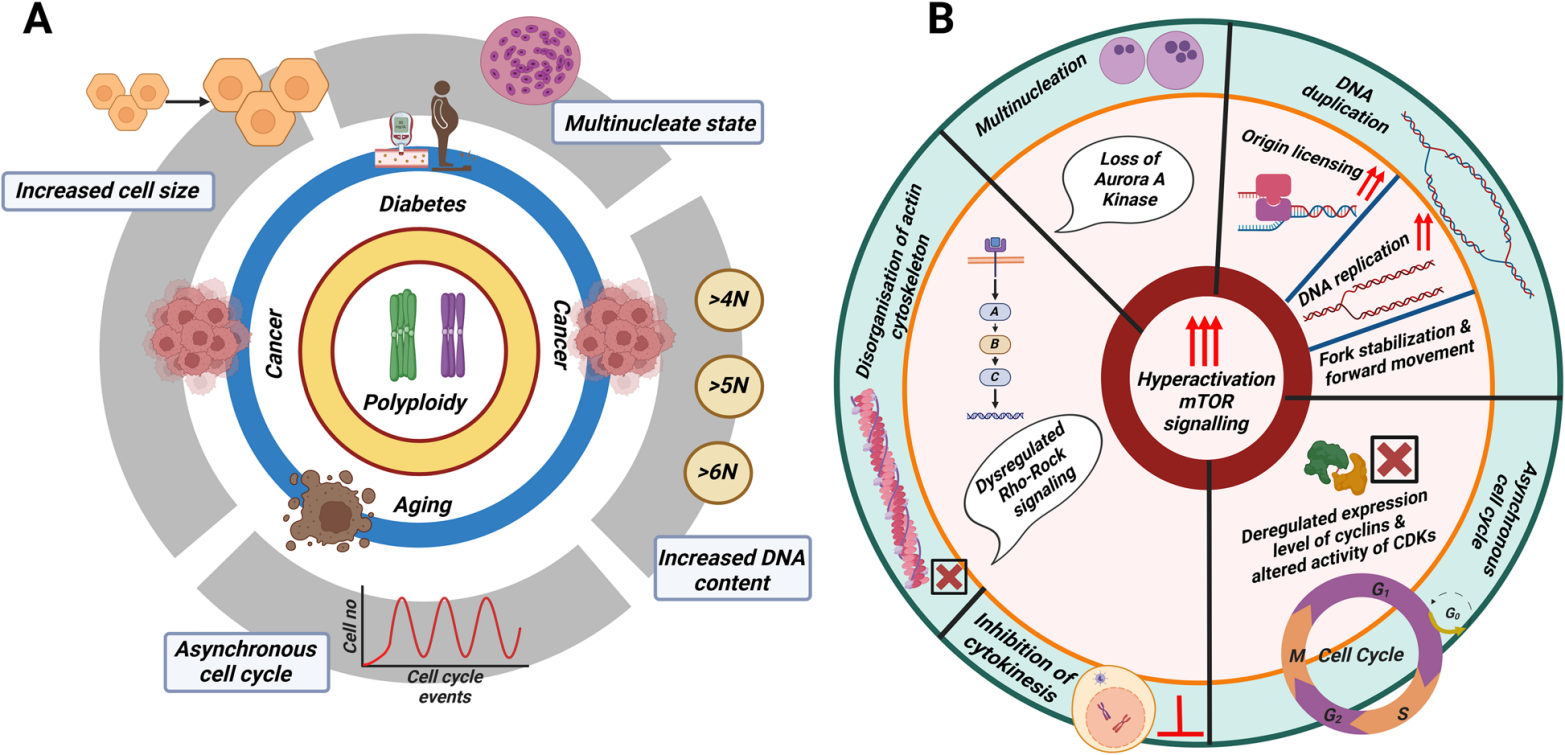
Association of the mTOR signaling pathway with hallmarks of polyploidy:(**A**) Depiction of the various hallmarks of polyploid cells: Hallmarks of polyploid cells include increased cellular size, multinucleation, increased DNA content and an asynchronous cell cycle. **B** Association of activated mTOR signaling with increased cellular size, multinucleation, increased DNA content, DNA duplication and various stages of the cell cycle

Many polyploid cells have characteristics that make them stand out. These features include (≥ 4N) DNA content, increased cellular size, multinucleated state and asynchronous cell cycle **(**Fig. 3A**)**.

#### DNA content (≥ 4n)

Flow cytometry has been utilized for DNA index (DI) analysis, thereby allowing measurement of DNA content in cells [72]. The majority of the polyploid cells in the adult *Drosophila* brain appears to be tetraploid, and the fraction of cells exhibiting ≥ 4n DNA content increases as the polyploidy increases [53]. Polyploidy refers to an increase in the overall amount of DNA in the cell's genome [73]. This occurs when one or more chromosomes are completely duplicated, which is indicated by number representations such as 4n, 5n and 12n [7]. Cancer cells with a relatively high DNA content usually appear as 4n + , 6n + or higher, and exist in the form of poly-aneuploidy [7]. In comparison to diploid cells, PGCCs exhibit a DNA content of tetraploidy or even higher abundance (≥ 4n) [74]. Numerous tumors exhibit the presence of cells with 4n, 8n or higher DNA content, and the presence of polyploid cells is widely acknowledged as an unfavourable prognostic marker across multiple types of cancer [17].

#### Increased cellular size

In comparison to diploid cells, PGCCs can exhibit a size that is 10 to 20 times larger [75]. Since polyploidy was first discovered, it has been postulated that larger cells play an important role in the physiological and developmental changes that come along with genome doubling, usually by changing the ratio of surface area to volume [76]. Polyploid individuals often develop larger bodies due to their increased cell size [77]. Nevertheless, the growth in size associated with ploidy is not linear or consistent [78]. The most naïve hypothesis for the increased cellular size due to polyploidy is that increases in gene copy number result in an increased amount of protein, which in turn increases cell volume [77]. Additionally, higher cell volume should be accompanied by increased two-dimensional membranes and cytoskeletal structures. While the augmented cell size of PGCCs is well recognized, the functional significance of increased cellular machinery has yet to be determined [7]. It is possible that the increased cell size allows PGCCs to store more energy molecules (e.g. lipids, proteins and carbohydrates) and to survive extended periods of dormancy [79]. It is postulated that increased cell size provides protection from toxin and oxidative stress via increased production of RNA and protein for protective pathways [79].

#### Multinucleated state

Polyploidy can arise from the presence of many nuclei resulting from failed cytokinesis or fusion of cells [6]. It applies to multinucleated cells wherein each individual nucleus contains two or more nuclei. Polyploidy and multinucleation are characteristic features of mammalian cardiomyocytes [58]. In a study on prostate cancer cells, administration of docetaxel chemotherapy in the cancerous cells reportedly leads to the formation of multinucleated polyploid cells [80]. Moreover, tumors and cell lines exhibit the presence of polyploid giant cells, which are characterized by substantially increased genomic content, and are frequently accompanied by the presence of multiple nuclei [81]. Cancer therapy resistance is associated with increased numbers of PGCCs, which are often multinucleated [81].

Melanocytes on exposure to UV suffer DNA damage and reactive oxygen damage. This damage results in multinucleation or polyploidy generation [81]. X-Ray irradiation of human glioblastoma multiforme cells results in the generation of multinucleated PGCCs [82]. These cells grow slowly and provide resistance to radiotherapy. Multinucleated polyploid cells significantly contribute to altering the composition of cancer genomes, and tumor evolution, and making them therapy-resistant [64]. The benefit of multinucleation in polyploid cancer cells is still unknown. One possible hypothesis suggests that the presence of multinucleated states in polyploid cancer cells likely helps in the evolution of tumor, thereby making the cells therapy-resistant [64]. This hypothesis holds since multinucleation in roundworms has been shown to provide evolutionary benefits.

#### Asynchronous cell cycle

In multicellular eukaryotes, mitosis is the recognised way for somatic cell to divide, making sure that duplicate genetic material is correctly split into progeny cells [83]. Eukaryotes are orderly developmental organisms that have carefully-controlled cell cycle resulting in low mutational frequencies [84]. Polyploid tumor cells have been shown to undergo aberrant mitosis [84]. Examination of several tumor tissue sections revealed the existence of proliferating cells having chromosomes of aberrant structure and size [85]. Polyploid cells show an asynchronous cell cycle, which suggests that all the nuclei in the polyploid cells are present in different phases of the cell cycle [86]. Cell cycle asynchrony is observed in polyploid neuroblasts. The staining of different nuclei within a polyploid cell with different colors showed that they are present in different phases of the cell cycle [84]. PGCCs derived from infection with cytomegalovirus showed an asymmetric, infrequent, or asynchronous cell cycle [64]. The asynchronous cell cycle promotes polyploid cell growth by either endomitosis or endocycle [4]. Cell cycle asynchrony results in generating genomic instability [86, 87]. Historically, it has been believed that polyploidy leads to chromosomal instability (CIN), ultimately culminating in tumorigenesis [86, 87]. This CIN might result from the asynchronous cell cycle in polyploid cells [86, 87]. Asynchronous cell cycles result in DNA damage. While DNA damage checkpoints are in charge of pausing the cell cycle in response to DNA damage, the genetic makeup of polyploid cells, which have an asynchronous cell cycle, allow them to enter mitosis [86]. Entry of cells with DNA damage into mitosis results in CIN ultimately aborting cytokinesis [88]. PGCCs’ asymmetric cell division by meiosis-like depolyploidization has previously been presented to explain their surprising life cycle [89]. The main objective of future research should be directed toward better understanding how PGCCs' asymmetric cell division affects PGCC development and function.

### mTOR signaling in cancer, diabetes and aging

It coordinates eukaryotic cell development and metabolism [13]. Over the past two decades, mTOR has been shown to govern several essential cell functions, and dysregulated mTOR signaling has been linked to cancer, diabetes and aging (Fig. 2) [13].

#### mTOR in cancer

mTOR has a close relationship with cancer given that it has implications in the control of several essential processes such as cell cycle, proliferation, growth and survival as well as protein synthesis and glucose metabolism (Fig. 2) [13]. mTOR signaling is one of the most commonly affected cascades in human cancers, and data in solid tumors showed that it is dysregulated in almost 30% of cancers [90]. Changes in the expression and activity of cancer-critical genes frequently cause mTOR signaling hyperactivation (Fig. 2) [91].

mTORC1 activation results in an increased protein synthesis due to repression of 4E-BP1 and activation of eIF-4E [13, 14]. eIF-4E activity induces an increase in the translation of pro-oncogenic proteins which in turn manage multiple processes such as cell survival, migration, and the growth of new blood vessels [92]. In addition, mTOR activation increases ribosomal biogenesis, thereby supplying the necessary tools to sustain high rates of cell development [93]. Metabolism of cancer cells appears to be reprogrammed to meet the needs of fast cell development. The mTOR complex has been portrayed as a nutrient receptor in cancer metabolism, in particular for nutrients such as glucose, amino acids, nucleotides, fatty acids and lipids as well as growth factors and other stressors [13].

Through the activation of S6K1, mTORC1 can also increase purine and pyrimidine synthesis which is crucial for DNA replication in cancer cells [94, 95]. Additionally, mTOR controls autophagy which breaks down and recycles cytosolic components in response to a lack of nutrition and energy [96]. The process of autophagy is frequently considered as a carcinogenesis inhibitor, and its suppression promotes the development of cancer **(**Fig. 2**)** [97, 98]. mTORC1 phosphorylates UNC-5-like autophagy-activating kinase 1 (ULK1), and prevents it from forming the ULK1-ATG13-FIP200 complex, thereby activating autophagy [99]. In contrast, mTORC2 can indirectly suppress autophagy by activating mTORC1 [100].

Many cancer oncogenes including LncRNAs, and splicing factors depend on mTORC1 [101–104]. For example, MALAT1 LncRNA acts as an oncogene in hepatocellular carcinoma (HCC) through the activation of mTORC1 signaling pathway [102]. Furthermore, it has also been shown to regulate the process of gluconeogenesis, a downregulated pathway in HCC, through mTORC1 [101].

In addition, mutations in mTOR itself have been identified in diverse subtypes of cancer, providing further evidence for the involvement of mTOR in the development of tumors [105]. The primary contribution of mTORC2 signaling to cancer is mainly due to its capacity to stimulate Akt, which facilitates pro-proliferative mechanisms such as glucose uptake and glycolysis, while simultaneously suppressing apoptosis **(**Fig. 2**)** [13]. Importance of mTORC2 signaling in cancer is further supported by the crucial function of Rictor, a vital core component of mTORC2, in mouse models of PTEN-loss-driven prostate cancer as well as in PTEN-deficient human prostate cancer cell lines [106].

#### mTOR in diabetes

Diabetes mellitus (DM) is a heterogeneous metabolic disorder of chronic hyperglycaemia [107]. Metabolic profiles of diabetic patients are highly disturbed, having increased level of glucose and lipids, causing the respective disorders of hyperglycaemia and hyperlipidaemia because of insulin resistance (Fig. 2) [108]. Hyperactivation of mTORC1 regulates insulin and growth factor signaling through insulin receptor substrates (IRS) [109]. mTORC1 has been linked to both Type 1 and Type 2 diabetes, implicating its involvement in the pathogenesis of these conditions [14]. Excessive and prolonged activation of mTORC1 leads to the inhibition of IRS via p70S6K (p70 ribosomal protein S6 kinase) [110, 111]. In consequence of this pathway, IRS loses its ability to facilitate the translocation of glucose transporters to the cell surface, ultimately resulting in elevated blood glucose levels and development of T2D. The activation of the hepatic mTORC1/S6k signaling pathway is responsible for the onset of hyperlipidaemia (Fig. 2) [112]. The consumption of excessive energy in the form of fats and proteins serves as the underlying factor in metabolic imbalances and metabolic disorders that contribute to obesity [112, 113]. Amino acids derived from dietary proteins enter the cytoplasmic circulation and actively participate in the activation of the mTORC1-p70S6K pathway through various signaling cascades [112]. The upregulation of the sodium-coupled neutral amino acid transporter (SNAT2) induces activation of the mTORC1-p70S6K pathway, resulting in elevated serum triglycerides (TGs) and decreased adipose lipoprotein lipase (LPL) expression (Fig. 2) [112]. The expression of dominant-negative p70S6K impedes the rise of hepatic triglycerides (TGs) in mice [112]. Prolonged activation of mTORC1 leads to insulin resistance, potentially exacerbating obesity and promoting the accumulation of lipid deposits (Fig. 2) [110, 111]. The intriguing association between the mTORC1-p70S6K pathway and lipid metabolism has attracted attention as it plays a significant role in the biosynthesis of fatty acids [114, 115]. mTORC1 is mandatory for de-novo lipid synthesis in murine liver [114, 115]. Fasting causes the liver to produce more ketone bodies (ketogenesis), which are then utilized by the distant organs as an energy source [116]. Hyperactivation of mTORC1 in the liver causes a noticeable defect in the production of ketone bodies (Fig. 2) [117]. The main transcriptional activator of ketogenic genes, peroxisome proliferator activated receptor (*PPAR*) is increased at the time of fasting [116, 118]. This procedure requires mTOR inhibition. By inhibiting PPAR activity, mTORC1 regulates ketogenesis [119].

Increased lipid synthesis occurs as a result of the upregulation of sterol regulatory element-binding protein 1c (SREBP1c) [120]. The function of SREBP1 is eliminated when mTORC1 is specifically inhibited in the liver [121]. Hyperinsulinemia emerges as a key factor that contributes to hepatic insulin resistance and the development of steatosis (accumulation of lipids) [122]. The inability of insulin to effectively act on skeletal muscle and the liver results in elevated blood glucose levels, leading to hyperglycemia. Prolonged hyperactivation of mTORC1 is associated with at least three distinct outcomes. As for the first outcome, IRS stops responding to insulin signaling, leading to high blood glucose level and consistent production of glucose in liver cells [123, 124]. The second outcome is the development of hyperlipidemia and hypertriglyceridemia, which contribute to insulin resistance and excessive hepatic glucose production, eventually leading to the accumulation of fatty acids and lipid deposits within cells **(**Fig. 2**)** [115, 125]. The third outcome involves inter-tissue communication leading to a reduction in lipoprotein lipases in the bloodstream, resulting in elevated triglyceride levels [112]. mTORC2 controls glucose and cholesterol homeostasis via AKT signaling [114, 126]. Insufficient mTORC2 expression in the liver causes faulty insulin-stimulated AKT phosphorylation, constitutive gluconeogenesis, impaired glycolysis and impaired lipogenesis via altering hepatic glucokinase and SREBP1c activity [127]. The regulation of gluconeogenesis and lipogenesis by mTORC2 has also been linked to a variety of transcription factors including FOXO1, FOXA2 and PPARγ [114, 127].

#### mTOR in aging

mTOR signaling is strongly implicated in the aging process of diverse organisms including yeast, worms, flies and mammals (Fig. 2) [13, 16]. The initial observations regarding the extension of lifespan were made in the nematode *C. elegans*, where it was discovered that decreased expression of the mTOR homolog (ceTOR, formerly let-363) or Raptor homolog (daf-15) is associated with increased longevity [128, 129]. Subsequent genetic studies found that reduced mTOR signaling also promotes longevity in *Drosophila* [130], budding yeasts [131] and mouse models (Fig. 2) [132, 133]. In line with these findings, rapamycin, an inhibitor of mTORC1, has demonstrably shows the unique ability to increase lifespan in various model organisms, making it the sole pharmacological intervention known to extend lifespan across different species [134–137]. Caloric restriction (CR), which refers to a decrease in nutrient intake without compromising nutritional requirements, is the only alternative intervention known to extend the lifespan across a diverse range of organisms [13]. Given the critical role of mTORC1 in sensing nutrients and insulin, many speculated that the beneficial effects of CR on life span are due to reduced mTORC1 activity [13]. Several lines of evidence suggest that the general reduction in mRNA translation during mTORC1 inhibition slows aging by reducing the accumulation of proteotoxic and oxidative stress, consistent with the observation that loss of the mTORC1 substrate S6K1 also extends life span in mammals (Fig. 2) [138]. A related possibility is that inhibition of mTORC1 slows aging by increasing autophagy, which helps clear damaged proteins and organelles such as mitochondria, the accumulation of which is also associated with aging and age-related diseases [13]. Finally, another model suggests that the attenuation of adult stem cells in various tissues plays a central role in organismal aging, and mTOR inhibition boosts the self-renewal capacity of both hematopoietic and intestinal stem cells in mice (Fig. 2) [139, 140]. mTORC1 signaling in aging stems cells regulates a diverse array of critical cellular processes [13], and its inhibition can increase lifespan and delay age-related ailments in mammals.

### Correlation of hallmarks of polyploidy with mTOR signaling

There exists a strong correlation between hyperactivation of mTOR signaling and hallmarks of polyploid cells like (≥ 4N) DNA content, increased cellular size, multinucleated state and asynchronous cell cycle (Fig. 3).

#### Multinucleation and mTOR signaling

The addition of maca (a plant product) to skeletal muscle cell culture has been shown to drastically enhance multinucleation in comparison to the control group via activating muscle hypertrophic signaling pathways such as Akt and mTOR [141]. Polyamines stimulate the formation of multinucleated trophectoderm cells that give rise to giant cells in the placentae of mice [142]. Polyamines have also been shown to activate mTOR signaling [143]. mTOR signaling has been found to be associated with cell multinucleation in hyperplastic skin that has undergone epidermal loss of AURORA-A Kinase [144]. Ectopic miR-100 expression in the MCF-7 luminal A cell line enhances the effect of paclitaxel on multinucleation by targeting the mTOR pathway [145]. Skeletal muscle hypertrophy is characterized by multinucleated fibers [146]. Moreover, hypertrophy is also characterized by activation of the mTOR signaling pathway [147]. All these studies clearly suggest that there is a distinct association between multinucleation and mTOR signaling (Fig. 3B).

#### Cell size and mTOR signaling

The IGF/PI3K/AKT/mTORC1 pathway is the best-known regulatory pathway that regulates cell growth [13]. It has been established that this evolutionary conserved pathway is a crucial regulator of cell growth, and consequently, a crucial factor in determining cell size **(**Fig. 3B**)**. In addition, artificial activation of this pathway results in increased growth in most of the examined cell types. The increased cell size observed in muscle cells during hypertrophy is associated with an increased mTORC1 signaling pathway [148]. Multiple signaling pathways are activated when insulin like growth factor (IGF) binds to its receptor, but the activation of the PI3K/AKT/mTORC1 axis with mTORC1—a critical mediator of the signal from the growth factor to biogenic pathways—is crucial for controlling cell growth and cell size [149, 150]. Additionally, mTORC1 functions as a signaling node at which energetic and stress signals can modulate growth factor signaling by integrating inputs from at least four additional key cues that can affect cell growth and cell size: stress, energy status, oxygen levels and amino acid levels [150, 151]. Cell size reduction has been induced by inhibition of the mTOR/S6K-signaling in Jurkat cells [152]. The nutrient-activated mTORC1 signaling system regulates protein synthesis, ribosome biogenesis, mRNA translation and autophagy to regulate cell size [13, 150, 151]. Vimentin is an intermediate filament protein in the cytoskeleton that controls cell size through mTORC1 signaling as observed in mouse model [153]. It has further been reported that vimentin has crucial implications in the progression of cancer and wound healing [154].

####  > 4N DNA content and mTOR signaling

mTOR signaling is crucial for controlling DNA duplication at various cell cycle phases (Fig. 3B) [155]. First, mTOR regulates CDC6 to govern DNA replication origin licensing [156]. Second, mTOR helps DNA replication forks move forward by keeping the levels of CDC6 and ribonucleotide reductase steady [155]. Third, by boosting the expression of CHK1 and FANCD2, mTOR keeps the replication fork stable [155]. It has been reported that replication stress may aid in the recruitment of mTOR to stalled replication forks [157].

#### Cell cycle and mTOR signaling

The mTOR signaling pathway participates in the control of translational initiation and early G1 progression in response to nutrient availability (Fig. 3B) [13]. Treatment with rapamycin results in the accumulation of the cyclin-dependent kinase inhibitor p27 in the cells, leading to cell cycle arrest in the G1 phase [158]. In mammals, and likely in many other classes of organisms, mTORC1 promotes progression of cells from G1 into S-phase*,* for example, by regulating the levels of specific cyclins and thus the activity of CDKs (Fig. 3B) [159]. Phosphopeptide mapping and mutational analysis have shown that raptor’s phosphorylation during mitosis is a key part of moving the cell cycle through the G2/M phase (Fig. 3B) [160]. mTORC1 regulates cytokinesis through the activation of Rho-ROCK signaling (Fig. 3B) [161]. Accumulating evidence indicates that one of the primary functions of mTORC2 is actin cytoskeleton rearrangement [162]. Deletion of mTORC2 disrupts the polarized organization of the actin cytoskeleton [162]. mTORC2 has been proposed to control the actin cytoskeleton through the activation of Rho GTPases [163]. The network of actin filaments plays a crucial role in regulating cells' cytoskeleton, which needs to undergo dynamic tuning and structural changes in order for cell division to take place in live cells [164]. Actin is a prominent regulator of cell division, a process whose success directly depends on the morphological changes of the actin cytoskeleton and the correct segregation of duplicated chromosomes [164]. Disorganization of the actin framework during the last stage of cell division, known as cytokinesis, can lead to multinucleation and the formation of polyploidy in post-mitotic cells [164]. During mitosis, this actin cytoskeleton undergoes reorganization, leading to the formation of rounded cells [164]. Following mitosis, the actin cytoskeleton is re-established, enabling cells to regain their elongated shape [165]. The cytokinesis event requires the highly coordinated reorganization of the cytoskeleton [165].

#### Polyploidy, senescence and mTOR signaling

**Fig. 4:**
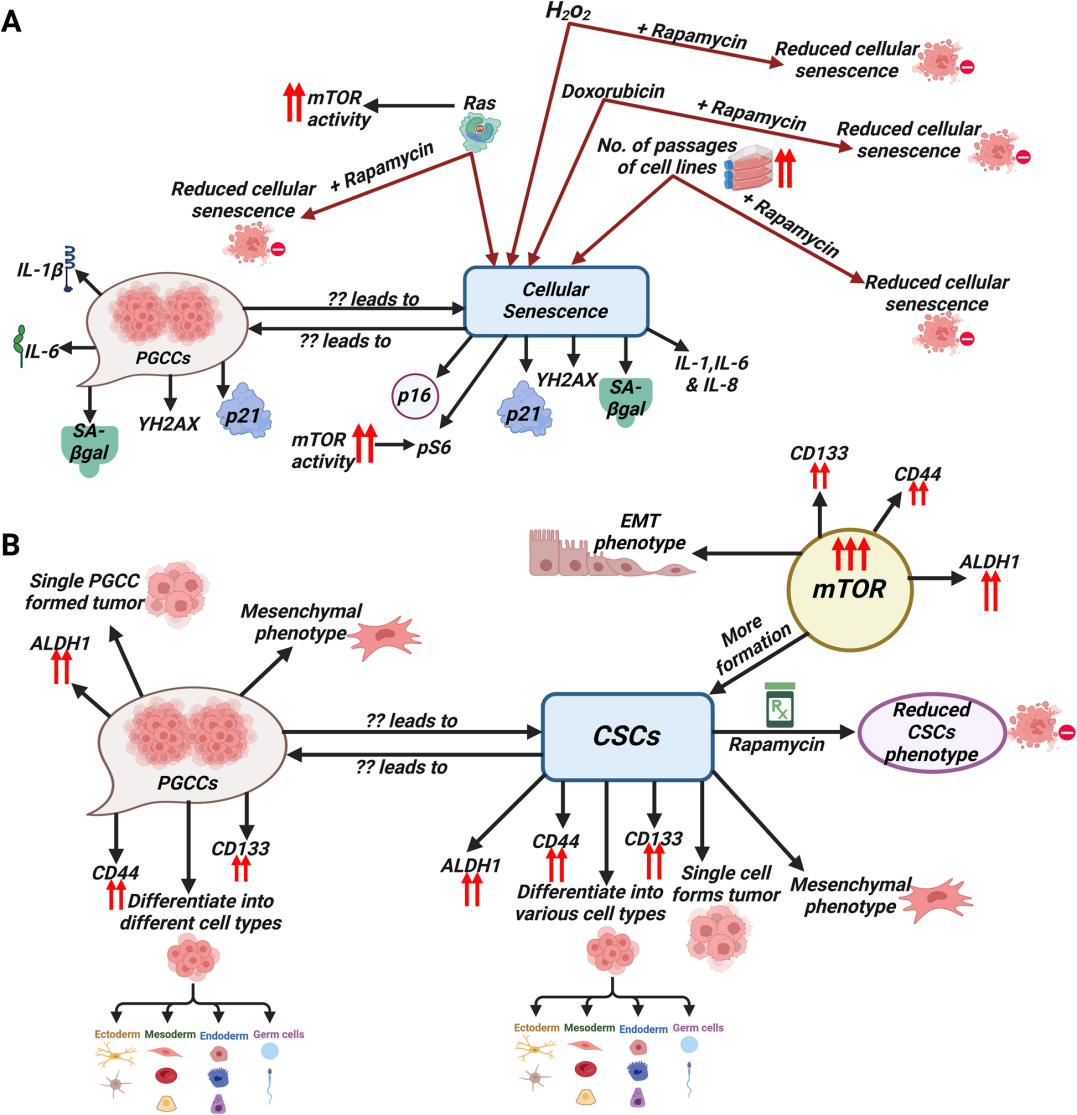
The molecular connection between the mTOR signaling pathway and polyploid giant cancer cells: (**A**) The interplay between polyploidy, senescence and mTOR signaling. **B** The interplay between polyploidy, stem cells and mTOR signaling

PGCCs exhibit hallmarks of cellular senescence (Fig. 4A) [166]. Features such as enlarged size and flattened morphology in PGCCs are suggestive of senescence in these cells [166]. Senescent phenotype is often characterized by multiple events such as induction of γ-H2A histone family member X (γ-H2AX) nuclear foci, cell cycle arrest regulated by the cyclin-dependent kinase inhibitors p16INK4a and p21, increase of senescence-associated β-galactosidase (SA-β-gal) activity, and enhanced expression of cytokines, namely interleukin-1 (IL-1), IL-6, and IL-8 [167–170]. PGCCs exhibit marked intensity in staining in SA-β-gal staining in the cytoplasm, whereas PGCC progeny cells express less β-gal than do the parental PGCCs [166]. In addition, γ-H2AX foci and p21 expression are highly enhanced in the nuclei of PGCCs and dropped to an undetectable level in the progeny cells [166]. Furthermore, the levels of IL-1β and IL-6, two components of the SASP are significantly elevated in PGCCs [166]. Gene set enrichment analysis from RNA sequencing analysis of whole transcriptomes indicated that cytokines and chemokines associated with the SASP phenotype, such as increased tumor necrosis factor-α signaling and cytokine activity, are enriched in the PGCCs [166]. These cumulative findings clearly suggest that PGCCs display several major hallmarks of cellular senescence (Fig. 4A).

Emerging data suggest that there is a definite role for mTOR in promoting cellular senescence [171]. For example, in a human fibrosarcoma cell line, HT-p21, simultaneous stimulation of growth and inhibition of the cell cycle results in cellular senescence [172]. However, treatment with rapamycin results in diminished cellular senescence, clearly indicating the role of mTOR in promoting senescence [172]. Supporting this idea, in another normal human fibroblast, WI-38, rapamycin treatment prevents or attenuates senescence induced by the chemotherapy drug doxorubicin (DOX) [172]. In addition, rapamycin has been shown to decrease hydrogen peroxide (H_2_O_2_)-induced senescence in ARPE-19 cells (a human retinal pigment epithelial cell line) [173]. Apart from chemical-induced senescence, rapamycin has been shown to slow down senescence in a number of other situations, including replicative senescence and oncogene-induced senescence (OIS) [174–176]. A study on replicative senescence of human skin fibroblasts (BJ cells) found that rapamycin treatment for three consecutive days stop the production of IL-8 and p21 [175]. Upon 60 passages, BJ cells show a senescence-associated phenotype, which is markedly decreased in rapamycin-treated BJ cells [175]. In the case of OIS, which is induced by the oncogene RAS, rapamycin-treated cells show a reduced senescence phenotype compared to non-treated cells [175].

An important sign that shows mTORC1’s involvement in cellular senescence is that S6 phosphorylation is higher in senescent cells, which is a well-known indicator of mTORC1 activity [176]. Furthermore, mTOR inhibition by rapamycin treatment attenuates the activation of some, if not all, senescent markers [176]. These data collectively suggest an intriguing role of mTOR in establishing cellular senescence (Fig. 4A). A variety of oncogenic proteins, such as RAF and RAS, known well to cause cellular senescence, can activate the mTOR pathway [177, 178].

#### Polyploidy, stem cell and mTOR signaling

PGCCs, which have cancer stem cell (CSC)-like characteristics, are known to express CSC-related markers such as CD44 and CD133 [30]. PGCCs can rapidly produce small-sized progeny cells through asymmetric divisions [30].

PGCCs that are isolated and cultured from human ovarian cancer cell lines and primary ovarian cancer show features of both normal and cancer stem cells [30]. These giant cells split up unevenly at the time of cell division, and show slow progression in terms of the cell cycle [30]. They are able to differentiate into other types of cells as well [30]. A single PGCC, like CSE, has been shown to form cancer spheroids in vitro and generate tumors in immunodeficient mice [30]. A PGCC-derived cell is shown to have a mesenchymal phenotype with increased expression of the CSE markers CD44 and CD133, and resistant to the treatment of cisplatin [30]. It was also shown that PGCCs made from human mammary epithelial cells (HMECs) that are infected with a human cytomegalovirus have the traits of stem cells, and the ability to change between epithelial and mesenchymal cells. The PGCC progenies resemble blastomeres during embryonic development, and can differentiate into different cell types in vitro [179]. They also show changes in the levels of expression of markers for embryonic development and self-renewal like NANOG, OCT3, OCT4, ALDH1A and SOX-2 [179]. Together, these findings reveal that the PGCCs possess normal and cancer stem cell-like properties (Fig. 4B) [30, 179].

The mTOR signaling pathway has a role in promoting the transition of ovarian cancer cells from an epithelial to a mesenchymal state [180]. Stimulation of the mTOR signaling pathway also amplifies the movement and infiltration of CSCs in prostate and pancreatic cancers [181, 182]. The inhibition of PTEN leads to the activation of mTORC1, which in turn enhances survival propensity, preservation of stem cell characteristics, and ability to form tumors in CD133 + /CD44 + prostate CSCs [183]. Activation of the mTOR signaling pathway stimulates cell proliferation, migration and invasion in head and neck squamous CSCs that express high levels of ALDH and CD44 markers of stem cells [184]. The activation of mTOR enhances the viability and reproduction of breast CSCs and NPC stem cells [185, 186]. Activation of mTORC1 also enhances the activity of aldehyde dehydrogenase 1 (ALDH1) in colorectal CSCs [187]. Activation of mTORC2 increases the expression of EpCAM, a hallmark of hepatic CSCs, and enhances their ability to form tumors in the liver [188]. Furthermore, a strong correlation between the mTOR signaling system and the metabolic processes of CSCs has been established [189]. For instance, deficiency of folate (LF) aids in a reprogramming of metabolic signals by activating the mTOR signaling pathway. This activation in turn promotes the spread and ability to form tumors of lung CSCs [189]. mTOR inhibitor, hinders the growth of breast CSCs by specifically targeting mitochondrial metabolism, glycolysis and several other signaling pathways [190]. Findings from these studies strongly suggest a connection between the mTOR pathway and CSCs (Fig. 4B).

## Future directions and perspectives

### mTOR signaling and polyploidy

**Fig. 5:**
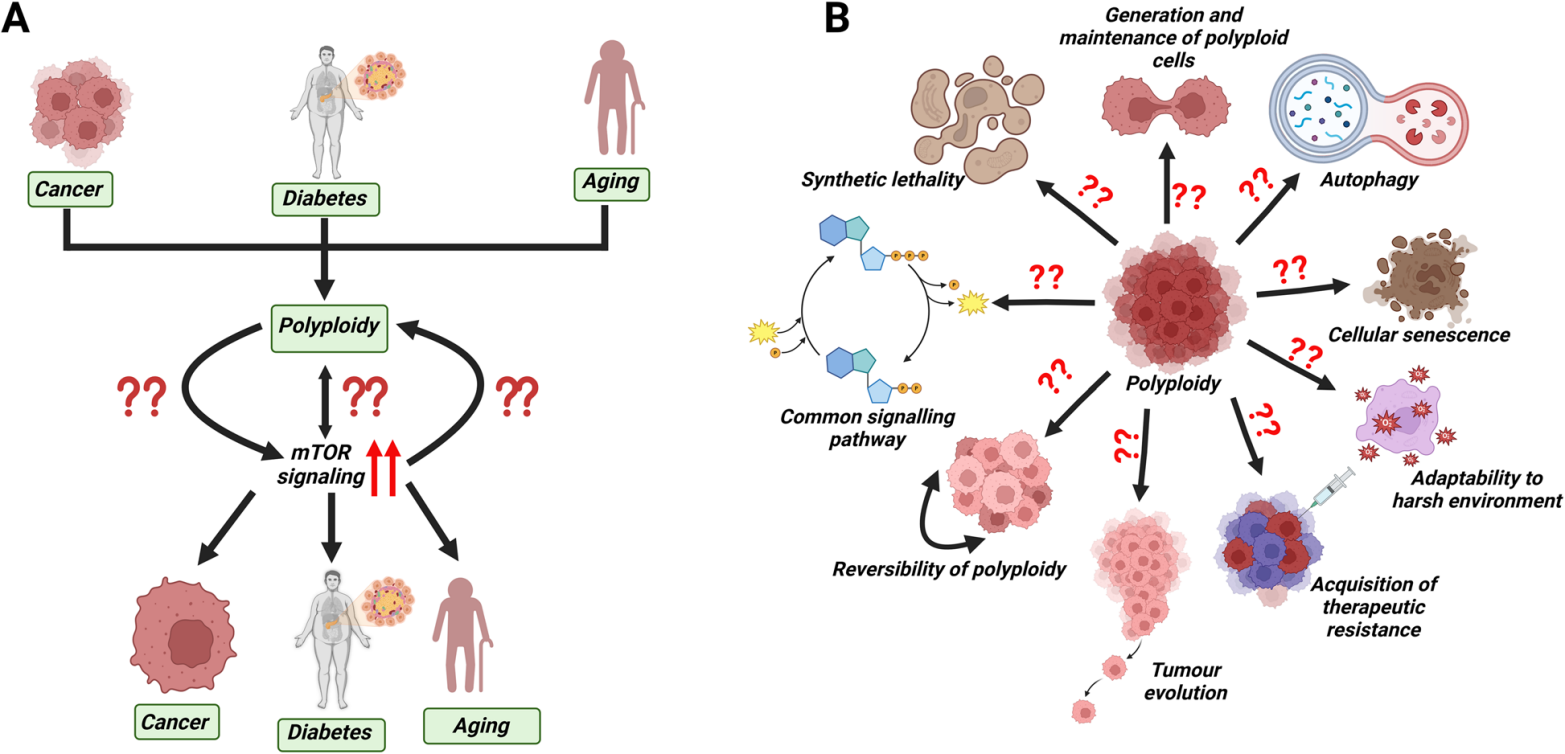
Prospective research directions in molecular interrelation between mTOR signaling and polyploidy: (**A**) The established links between polyploidy and mTOR signaling individually with cancer, diabetes and aging are well documented. However, the molecular connection between polyploidy and mTOR signaling remains to be elucidated. **B** Schematic illustration of the envisaged future research endeavors aimed at uncovering the intricate molecular association between polyploidy and mTOR signaling

While polyploidy and mTOR signaling separately are associated with cancers, diabetes and aging, the molecular connection between the two is highly likely. However, it remains to be answered whether polyploidy leads to the activation of mTOR signaling or vice-versa. Immediate investigation is required in order to address these questions of high importance **(**Fig. 5A). Further study is needed to explore whether mTOR signaling is essential for the propagation of polyploid cells as well. A common signaling pathway in association with polyploidy is yet unknown (Fig. 5A). Research based knowledge on the cause and consequences of polyploidy has the potential to be therapeutically exploited for the treatment of cancer, diabetes and aging. The cell signaling mechanism that controls the inception of polyploidy, and could serve as a biomarker needs investigation. With this regard, mTORC1 signaling has huge potential.

Polyploid tumor cells were thought to assist tumors undergo quick changes, and thereby become resistant to treatment as observed in a number of cancers that do not yet have a cure for [191]. The significance of mTOR signaling in tumor evolution (Fig. 5B) or in the therapy resistance of PGCCs needs investigation. Polyploidy seems to provide some sort of adaptability to cancer cells in order to survive harsh conditions such as low serum, hypoxia and drugs [20]. The role or fate of mTOR signaling when polyploid cells are exposed to harsh environmental conditions needs investigation (Fig. 5B).

### Reversibility of polyploid cells

The capacity for evolutionary adaptation is a fundamental characteristic of biological systems [192]. Yeast cells deleted of cytokinesis genes have been shown to rapidly evolve divergent pathways to restore growth and cytokinesis [193]. Common genetic alterations associated with the best evolved strains are polyploidy and aneuploidy [193]. The fate of polyploid cells when they are transferred to normal conditions is unknown. An important question is whether polyploid cells revert to normal cells or remain polyploid when given favorable environmental condition. The role of signaling pathways, or the mTORC1 pathway in particular, in the conversion of polyploid cells requires investigation (Fig. 5B).

Rather than being considered a cell type with no future, PGCCs have their own life cycle and are now being considered to play an important role in the immortality, invasion, origin, metastasis and resistance of tumor cells to radiotherapy and chemotherapy [7]. The initial enthusiasm for the so-termed therapy-induced senescence gradually turned into a concern that senescent PGCCs might do more harm than good, and could lead to cancer recurrence [194]. One particular concern is the senescence-associated secretory phenotype (SASP) of PGCCs, which is the potential culprit in the detrimental effects of cancer cell senescence [195]. It seems likely that a fundamental role in this process is played by the reversibility of proliferation arrest, which is frequently connected with coupling of cell senescence with polyploidization or depolyploidization [195]. This study further suggests that progeny resulting from the reversibility of the polyploid state may be highly aggressive, leading to the formation of resistant disease and tumor recurrence. The role of polyploidy in senescence or the mechanism how senescent polyploid cells revert to normal cells as of yet is unknown **(**Fig. 5B**)**. Cellular senescence is frequently accompanied by the synthesis of secreted proteins that facilitate various impacts of senescence on the surrounding tissue microenvironment [56, 194]. mTORC1, a key controller of protein synthesis, has been demonstrated to regulate the senescence-associated secretory phenotype by influencing gene transcription, mRNA translation and -stability [173–176]. The role played by the mTOR signaling pathway in polyploidization or depolyploidization, or the significance of mTOR signaling in the SASP of polyploid cells, is yet to be deciphered.

### Generation and maintenance of polyploid cells

An extensive correlation exists between the generation of polyploid cells and a variety of cellular stressors [196]. Catastrophic DNA replication is observed in polyploid cells [197]. Conditions that propel polyploidy to promote cancer or inhibit tumor growth require investigation. Genomic instability and chemoresistance can give rise to cancer cells due to a unique form of plasticity that is observed in the PGCCs [20]. These giant cells emerge in response to chemotherapy-induced stress and exhibit chromosomal content exceeding the diploid level. The role of mTOR signaling in deciding the fate of polyploid cells is yet to be elucidated (Fig. 5B). Investigation is required to explore whether the conditions that accompany PGCCs to provide benefits or cause catastrophe to cancer cells are involved with the extent of polyploidy or the strength of mTOR signaling.

Genomic instability and chemoresistance can arise in cancer due to a unique form of plasticity of PGCCs [81, 198]. These cells form under the stress of chemotherapy and have a higher than diploid chromosome content [81]. PGCCs have been observed in ovarian cancer histology, including the deadly and common form of high-grade serous ovarian carcinoma (HGSC) [199]. It has been previously found that drugs that disrupt the cellular recycling process of autophagy are uniquely efficacious in pre-clinical HGSC models [199]. Generally, autophagy inhibitors sensitize cells to nutrient-depletion-induced cell death. A study used carboplatin or docetaxel to treat CAOV3 and OVCAR3 ovarian cancer cell lines, and observed an increased abundance of PGCC [199]. The autophagy inhibitors were tried to assess their efficacy on PGCC generation and maintenance [199]. However, contrary to the expectations, these inhibitors fail to hinder the formation of PGCCs in OVCAR3 or CAOV3 cells [199]. Interestingly, administering the mTORC1 inhibitor rapamycin surprisingly prevents PGCC colony outgrowth (52–84% inhibition) [199]. The detailed molecular mechanism of the role of mTORC1 in the generation and maintenance of polyploid cells needs further investigation.

The presence of dormant cancer cells that persist despite anticancer therapy can result in cancer recurrence and the development of metastasis, which often leads to fatal outcomes [198]. A study by You et al. highlighted the crucial role of autophagy in the induction of dormant PGCCs [200]. Furthermore, stopping autophagy either with drugs or by genetic modifications markedly slows down the formation of dormant PGCCs. This leads to a significant reduction in metastasis and increased survival in a mouse model [200]. The mechanism underlying PGCC formation involves partial damage to mitochondria by chemotherapeutic drugs, leading to decreased ATP levels. This in turn sets off autophagy through the AMPK-mTOR pathway, which ultimately promotes the formation of PGCCs [200]. The study elegantly showed that dormant PGCCs activate autophagy through downregulation of the mTOR pathway and activation of the AMPK pathway. The mTOR and AMPK pathways have opposing functions. Dormant PGCCs are induced due to the activation of autophagy, but the fate of polyploid cells under autophagic conditions is yet to be uncovered **(**Fig. 5B**)**.

### Glucose starvation and polyploidy

One of the autophagic conditions is glucose starvation. Every normal cell in the body uses blood sugar (glucose) for energy. Cancer cells use more glucose than normal cells [201]. The results in clinics of targeting cancer cells with glucose starvation have not been promising. The sensitivity to sugar deprivation varies among different types of cancer cells; even in the susceptible cases, the impact has been found to be limited to a slowdown of the cancer progression [202]. In cell culture, glucose starvation is one of the major forms of metabolic stress experienced by cancer cells. Under glucose starvation, the 5' AMPK activated protein kinase (AMPK) plays a critical role in maintaining redox homeostasis and cell survival [203]. The mTORC1 pathway has been shown to play an important role in controlling autophagy upon glucose starvation [204]. Since there is a high prevalence of polyploid cells in tumors and activation of the mTORC1 pathway is expected in polyploid cells, detailed investigation is required to unravel the molecular interplay between polyploidy and mTOR signaling in response to glucose starvation. It could be possible that polyploid cells experiencing glucose starvation, and the polyploid cells surviving glucose starvation would have different genetic make-ups. The polyploid cells might require mTORC1 activation for their generation and propagation, while dormant polyploid cells require activation of the AMPK pathway and downregulation of the mTORC1 pathway. Polyploid cells have been shown to consume more glucose and glutamine as compared to normal cells [205, 206]. Glucose starvation and glutamine depletion have been shown to reduce mTORC1 signaling [207, 208]. Theoretically, this implies that glucose starvation and glutamine depletion would reduce mTORC1 signaling, which in turn could reduce the propagation of polyploid cells.

### Synthetic lethality

When attempting specific targets on polyploid cells, use of ploidy-specific lethality as the target molecule could be a strategy worthy of consideration. Ploidy-specific lethality has been referred to a deletion of budding yeast gene that results in the death of polyploid cells although it lacks lethality in isogenic haploid or diploid cells [192]. Furthermore, it would also be interesting to identify analogous mammalian genes that are not required for the viability of cells with normal ploidy but are essential for the survival of cells with increased ploidy. Systemic study is required to determine whether these genes might play crucial roles in the survival of tumor cells that exhibit heightened levels of ploidy. Detection of drugs that specifically target these gene products could be useful as chemotherapeutic agents (Fig. 5B). Effective cancer therapeutics must be based on the physiological differences between cancer cells and normal cells. One possibility is that polyploid cells, during their progression, might constantly change their genetic makeup. Therefore, identifying the status of mTOR signaling in different stages of polyploid cells could be a future approach toward targeting polyploid cells.

## Conclusion

Recent advancements in genetics and signaling pathway-based research have underscored the pivotal role of polyploidy and the mTOR signaling pathway as major drug targets in modern medicine. Emergence of the roles of these components highlights their substantial involvement in the development and progression of cancer, diabetes and aging. Therefore, it is crucial to investigate the regulation of polyploidy in the context of disease progression and its response to therapy. In order to achieve this goal, it is crucial to collect large cohorts of tissue biopsies from individuals, coupled with their detailed clinical phenotyping. Analysis of these cohorts will aid in the exploration of the association between polyploidy and polyploidy-associated diseases.

Overall, we predict that the information gained by studying polyploidy and mTORC1 signaling would be translated and incorporated into future research toward designing novel anti-polyploidy therapies. Furthermore, understanding the molecular interplay between these pathways would likely be helpful to improve patient lifespan by slowing down the emergence of therapy resistance as well as picking suitable chemo-preventative agents in order to reduce the incidence of these diseases. Finally, polyploidy and the mTOR axis will help in better understanding the disease biology, which in turn would likely help devising potential strategies of treatment for polyploidy related diseases.

## Data Availability

No datasets were generated or analysed during the current study.
